# Interactive online application for the prediction, ranking and prioritisation of drug targets in *Schistosoma haematobium*

**DOI:** 10.1186/s13071-018-3197-6

**Published:** 2018-11-27

**Authors:** Andreas J. Stroehlein, Robin B. Gasser, Ross S. Hall, Neil D. Young

**Affiliations:** Melbourne Veterinary School, Department of Veterinary Biosciences, Faculty of Veterinary and Agricultural Sciences, The University of Melbourne, Parkville, Victoria 3010 Australia

**Keywords:** Drug targets, Computational drug discovery, *Schistosoma*, Prioritisation systems

## Abstract

**Background:**

Human schistosomiasis is a neglected tropical disease caused by parasitic worms of the genus *Schistosoma* that still affects some 200 million people. The mainstay of schistosomiasis control is a single drug, praziquantel. The reliance on this drug carries a risk of resistance emerging to this anthelmintic, such that research towards alternative anti-schistosomal drugs is warranted. In this context, a number of studies have employed computational approaches to prioritise proteins for investigation as drug targets, based on extensive genomic, transcriptomic and small-molecule data now available.

**Methods:**

Here, we established a customisable, online application for the prioritisation of drug targets and applied it, for the first time, to the entire inferred proteome of *S. haematobium*. This application enables selection of weighted and ranked proteins representing potential drug targets, and integrates transcriptional data, orthology and gene essentiality information as well as drug-drug target associations and chemical properties of predicted ligands.

**Results:**

Using this application, we defined 25 potential drug targets in *S. haematobium* that associated with approved drugs, and 3402 targets that (although they could not be linked to any compounds) are conserved among a range of socioeconomically important flatworm species and might represent targets for new trematocides.

**Conclusions:**

The online application developed here represents an interactive, customisable, expandable and reproducible drug target ranking and prioritisation approach that should be useful for the prediction of drug targets in schistosomes and other species of parasitic worms in the future. We have demonstrated the utility of this online application by predicting potential drug targets in *S. haematobium* that can now be evaluated using functional genomics tools and/or small molecules, to establish whether they are indeed essential for parasite survival, and to assist in the discovery of novel anti-schistosomal compounds.

**Electronic supplementary material:**

The online version of this article (10.1186/s13071-018-3197-6) contains supplementary material, which is available to authorized users.

## Background

Human schistosomiasis is a neglected tropical disease (NTD) caused by parasitic flatworms of the genus *Schistosoma* [1]. This disease affects some 200 million people, predominantly in Africa, and is mainly caused by two species, *Schistosoma mansoni* and *S. haematobium* [2, 3]. The mainstay of schistosomiasis control is a single drug, praziquantel, which, due to its low cost, effectiveness and safety, is being used widely in mass drug administration (MDA) programmes worldwide [4–6]. Arguably, the reliance on a single drug can increase the likelihood of selecting for resistant worms, given the knowledge of rapid and widespread resistance against all major drug classes in many parasitic worms of animals [4, 7]. Thus, research towards the discovery and development of alternative anti-schistosomal drugs, including the repurposing of drugs that are approved for use in humans, is on-going [4, 8, 9].

In this context, a number of studies (e.g. [10–12]) have employed computational approaches to prioritise proteins for investigation as drug targets, using extensive genomic and transcriptomic data now available for schistosomes [11, 13–15]. Similar approaches [16, 17] have been applied to other parasites of socioeconomic importance. Additionally, the online resource TDRtargets [18], although mainly established for unicellular pathogens, allows for the prediction of drug targets for a number of parasitic helminths that cause neglected tropical diseases [19, 20]. Another target prioritisation approach, which relies on both filtering and ranking of weighted gene/protein features, has been applied to predict kinases as drug targets in *Haemonchus contortus*, an economically important parasitic roundworm (nematode) [21]. However, this refined approach has, to our knowledge, not yet been applied to the protein complement of the flatworm *S. haematobium*. Here, we extend and enhance this approach by establishing an online application which allows for interactive weighting and ranking of features using parameters defined by the researcher. Using this application, we identified potential drug targets in *S. haematobium* that could be linked to approved drugs and inferred novel targets that are conserved among socioeconomically important parasitic flatworms, for which no known ligands exist.

In order to prioritise drug targets employing customisable prioritisation criteria, we first inferred sequence-based features using genomic and transcriptomic data, and then linked sequences to pathways and drugs. All of these features were then integrated into an online application.

### Transcription levels

To prioritise drug targets according to gene transcription in adult stages of *S. haematobium*, we recorded transcription levels for all 11,140 *S. haematobium* genes (PRJNA78265) from published data (accession numbers: SRR6655497 and SRR6655495) [11, 12, 22].

### Inferring orthologs in metazoan model organisms and *S. haematobium-*related flatworms

To determine amino acid sequence similarity between 11,140 individual *S. haematobium* proteins (PRJNA78265) and those of other species, we employed the program blastp v.2.2.28+ applying an E-value cut-off of 10^-5^ and considering only the best matches. We carried out these comparisons using protein sequences from WormBase (*Caenorhabditis elegans*, WS262, PRJNA13758) [23], FlyBase (*Drosophila melanogaster*, release FB2017_06, dmel_r6.19) [24], Ensembl (release 91.38; *Mus musculus* GRCm38.p5 and human GRCh38) [25] and WormBase Parasite (WBPS9; accession numbers: PRJEA36577, *S. mansoni*; PRJEA34885, *S. japonicum*; PRJDA72781, *Clonorchis sinensis*; PRJNA222628, *Opisthorchis viverrini* and PRJNA179522, *Fasciola hepatica*) [13–15, 26–28]. For each pair of query-hit sequences obtained from the blastp analysis, we then calculated a pairwise global alignment using the program NEEDLE from the EMBOSS package v.6.4, to determine global sequence similarity and coverage, which were used to define groups of orthologous sequences *via* the user interface of the online application.

### Inferring essentiality and functional annotation

Next, we inferred genes with a lethal phenotype in *C. elegans*, *D. melanogaster* and/or *M. musculus*, employing gene perturbation data and information from the WormBase, FlyBase and Ensembl databases, respectively, to allow for the prioritisation (i.e. selection or high weighting) of parasite orthologs with lethal phenotypes in one or more of these species. We then annotated *S. haematobium* proteins by matching them with their closest homologs in the Swiss-Prot [29] and the Kyoto Encyclopedia of Genes and Genomes (KEGG) [30] databases (releases 05/2017) using blastp, and by domain searches using InterProScan v.5.15.54 [31]. To prioritise proteins that represent pathway ‘choke-points’ (cf. [32–34]), we inferred proteins linked to a unique KEGG orthologous gene (KO) term within a KEGG pathway and/or annotated with a unique InterPro identifier.

### Predicting drug-drug target associations

To infer associations between parasite proteins and known drug targets, we matched *S. haematobium* proteins to known drug targets and associated drugs in the ChEMBL (release 23) [35] and DrugBank v.5-0-11 [36] databases using the program psiblast v. 2.2.26+, applying a stringent E-value cut-off of 10^-30^. For the ChEMBL database, we included only targets for which data from at least one drug screening assay were available, as otherwise no connection between a target (in a parasite) and a drug could have been made. In addition, we applied the following default selection criteria (cf. [37]): we only considered binding assays (“B”) that reported “activity” (in %), “inhibition” (in %), “Kd” (dissociation constant in nM), “Ki” (inhibitor constant in nM), “IC50” (half-maximal inhibitory concentration in nM) or “potency” (in nM). For all values reported in nM, we set the cut-off at ≤ 10,000 nM (10 mM) and, for all percentage values, we set the cut-off at ≥ 70%, in order to pre-filter the ChEMBL database for compounds that showed activity against an associated target and are thus predicted to have an effect on an orthologous protein in the parasite. Of these 109,712 compounds, we labelled 30,169 compounds as “previously screened in *Schistosoma* species”, as they were (in ChEMBL) either linked to *Schistosoma* as the test organism (*n* = 30,158) or had been screened against *S. mansoni* in a recent study (*n* = 11) [38]. In addition, we retrieved information from both databases on the clinical phase or status of approval of all compounds and the number of violations of the rule-of-3/rule-of-5 [39]. We also assessed whether small molecules in the ChEMBL database represented natural products and inferred the ‘drug-likeness’ of compounds in the DrugBank database based on their similarity to compounds from the MDL Drug Data Report (MDDR) [40].

### Online application to predict and prioritise drug targets in *S. haematobium* and to infer associated drugs

**Fig. 1:**
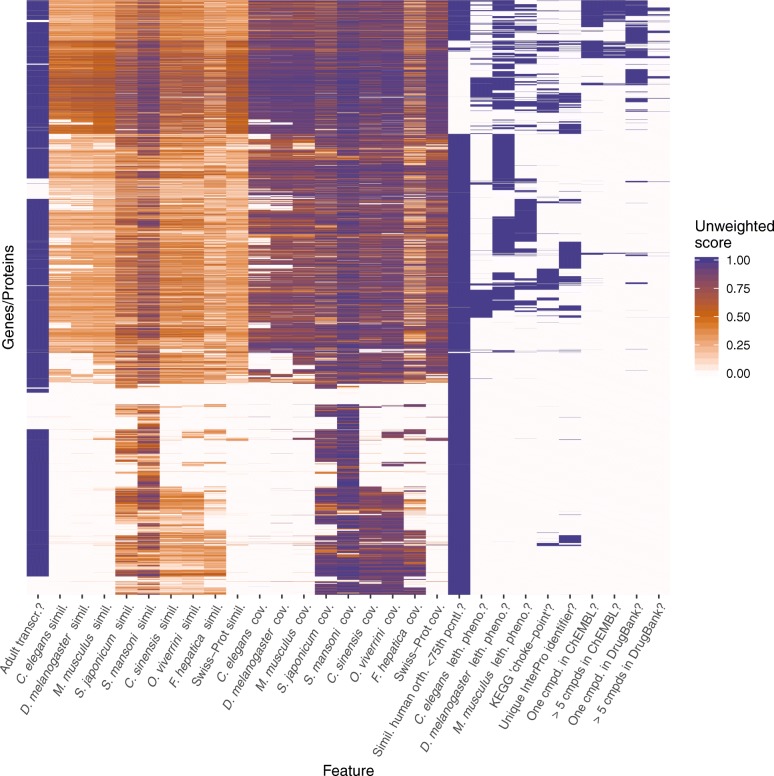
Distribution of all feature scores (unweighted) for *Schistosoma haematobium* genes/proteins (Table 1). Feature description ending with a “?” have an unweighted score of either “0” (“no”) or “1” (“yes”), whereas all other features are represented by percentage values of similarity or coverage, respectively. Proteins were clustered using the Ward clustering method based on the Euclidian distances between feature profiles of individual genes/proteins

**Fig. 2:**
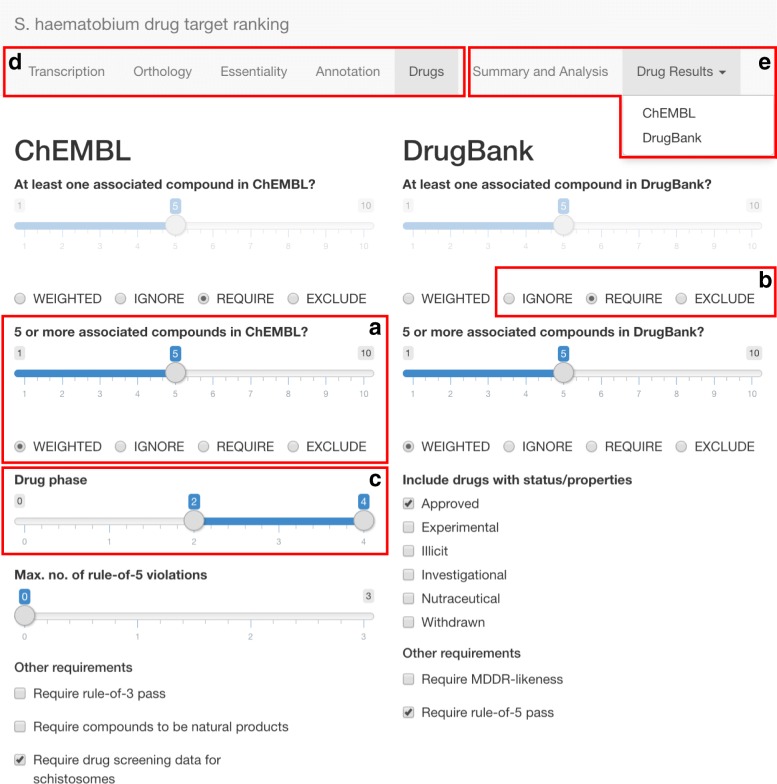
User interface of the online application. The weighting of features can be set *via* a slider (**a**) or features can be excluded, ignored or required (**b**). Additionally, feature restrictions/filters for associated drugs can be defined using a range slider (**c**) or check-boxes. Of the five panels that represent different steps in the ranking/prioritisation process (**d**) and the two panels that visually summarise and display the resulting proteins and drugs (**e**), the “Drugs” panel is shown here as an example

To enable the customisable prioritisation of drug targets based on all inferred target/compound properties, we developed an online application (available *via* [41]; source code available *via* [42]) using the *shiny* package [43] of the R programming language v.3.5.0, which allows for the inclusion of 29 gene/protein features (Table 1 and Fig. 1) in five different ways (Fig. 2). The first option is to assign a *weighting* to a particular property by means of a slider (Fig. 2a). The second option allows for a particular property to be *required* by selecting a check-box (Fig. 2b), thus removing (i.e. filtering) from the results all proteins/compounds that do not satisfy the required feature. The third option (Fig. 2b) *excludes* proteins that satisfy any feature that is deemed disadvantageous for a prospective drug target (e.g. high sequence similarity to a host ortholog). Using the fourth option, features that are (according to the investigator’s aims/priorities) deemed unimportant for the prioritisation process can be *ignored* (Fig. 2b). When this option is selected, no weightings are assigned to the features, regardless of whether a protein satisfies them or not. For some features, a fifth option allows for a *range* of values (e.g. clinical phase of a drug; Fig. 2c) or a *subset* of properties (e.g. a subset of InterPro domains of interest) to be selected. The final score for each protein that is not excluded from the results during the selection process is then determined by calculating the sum of weighting factors for all individual features. Subsequently, genes/proteins are ranked from highest to lowest overall scores, as described previously [21]. In the following, we describe and justify the weightings, parameters and cut-offs that we selected in the online application to prioritise drug targets in *S. haematobium*. The user interface of the application developed here consists of *five panels* (Fig. 2d) that represent the different steps in the prioritisation process as well as *two additional panels* (Fig. 2e) that visually summarise and display the prioritised proteins and drugs.

**Table 1:** Ranking and prioritisation features employed to infer drug targets and associated drugs in *S. haematobium*

Feature	Default (cut-off) value	Ranking type	Weighting
Transcribed in adult worms?	Yes	Require	na
Sequence similarity to *C. elegans* ortholog	≥ 80%	Weighted	5
Sequence similarity to *D. melanogaster* ortholog	≥ 80%	Weighted	5
Sequence similarity to *M. musculus* ortholog	≥ 80%	Exclude	na
Sequence similarity to *S. japonicum* ortholog	≥ 80%	Weighted	7
Sequence similarity to *S. mansoni* ortholog	≥ 80%	Weighted	8
Sequence similarity to *C. sinensis* ortholog	≥ 80%	Weighted	6
Sequence similarity to *O. viverrini* ortholog	≥ 80%	Weighted	6
Sequence similarity to *F. hepatica* ortholog	≥ 80%	Weighted	8
Sequence similarity to Swiss-Prot ortholog	≥ 80%	Weighted	7
Sequence coverage of *C. elegans* ortholog	≥ 50%	Weighted	5
Sequence coverage of *D. melanogaster* ortholog	≥ 50%	Weighted	5
Sequence coverage of *M. musculus* ortholog	≥ 50%	Exclude	na
Sequence coverage of *S. japonicum* ortholog	≥ 50%	Weighted	7
Sequence coverage of *S. mansoni* ortholog	≥ 50%	Weighted	8
Sequence coverage of *C. sinensis* ortholog	≥ 50%	Weighted	6
Sequence coverage of *O. viverrini* ortholog	≥ 50%	Weighted	6
Sequence coverage of *F. hepatica* ortholog	≥ 50%	Weighted	8
Sequence coverage of Swiss-Prot ortholog	≥ 50%	Weighted	7
Sequence similarity to human ortholog ≤ 75th percentile?	Yes	Require	na
Lethal phenotype for *C. elegans* ortholog?	Yes	Weighted	9
Lethal phenotype for *D. melanogaster* ortholog?	Yes	Weighted	9
Lethal phenotype for *M. musculus* ortholog?	No	Exclude	na
KEGG ‘choke-point’?	Yes	Weighted	8
Unique InterPro identifier?	Yes	Weighted	7
One associated compound in ChEMBL?	Yes	Require	na
More than five associated compounds in ChEMBL?	Yes	Weighted	5
One associated compound in DrugBank?	Yes	Require	na
More than five associated compounds in DrugBank?	Yes	Weighted	5

For each feature, the default value or cut-off value, the ranking type chosen in this work and the assigned weighting are given. For the inference of *de novo* drug targets (i.e. those without associated drugs), the last four listed features (i.e. those describing the number of associated compounds) were all set to “exclude”

*Abbreviation*: na, not applicable

#### Transcription of target genes (panel 1)

In the first panel, “Transcription”, genes can be filtered (i.e. “required” or “excluded”) or assigned a weighting according to their transcription level in at least one adult stage (male and/or female) of *S. haematobium*. For the present analysis, we selected the option “required”, as we considered target transcription in at least one adult stage to be a minimum requirement for a compound to have an effect against this target in the parasite within the human host.

#### Sequence comparisons of target genes with orthologs of other species (panel 2)

The panel “Orthology” allows for the prioritisation of targets based on their conservation among multiple ‘model’ organisms (*C. elegans*, *D. melanogaster* and *M. musculus*), flatworms related to *S. haematobium* (*S. japonicum*, *S. mansoni*, *C. sinensis*, *O. viverrini* and *F. hepatica*), as well as orthologs that have been experimentally verified (i.e. Swiss-Prot orthologs) and host (i.e. human) orthologs. This panel also allows for the selection of cut-offs used to define which *S. haematobium* proteins have orthologs in other species. Here, we selected a minimum of 80% amino acid sequence similarity across the entire sequence and a minimum of 50% of sequence coverage as the cut-off for proteins to be recorded as orthologs.

Although proteins conserved across a broad range of taxa are suggestive of being essential [16], this characteristic might also indicate that the parasite protein cannot be safely and selectively targeted by a small molecule, which could lead to adverse or toxic effects when the drug also targets an ortholog in a host animal (vertebrate). Thus, here, we selected a weighting of five points for the presence of both *C. elegans* and *D. melanogaster* orthologs and excluded any targets that had an inferred ortholog in *M. musculus*. For the same reason, we required that there was no human ortholog and that any existing homologs were ≤ 46.2% (75th percentile) similar to the target sequence. Furthermore, we gave seven and eight points for the presence of orthologs in *S. japonicum* and *S. mansoni* respectively, given that *S. haematobium* and *S. mansoni* are often co-endemic [1, 2] and a novel anthelmintic should be able to target all species. In addition, we awarded six points to targets with orthologs in two other flatworm species (*C. sinensis* and *O. viverrini*) and eight points to targets with an ortholog in the liver fluke *F. hepatica*, given the major socioeconomic importance of this parasite [44, 45]. An ortholog in the Swiss-Prot database was given seven points, reasoning that an experimentally verified and/or manually curated ortholog provides additional confidence to the accuracy of the annotation of a parasite protein.

#### Inference of essentiality (panel 3)

In the “Essentiality” panel, we gave nine points to targets with *C. elegans* and *D. melanogaster* orthologs that exhibited lethal phenotypes upon perturbation by RNA interference (RNAi) of their respective genes. We excluded any targets that had an ortholog in a representative vertebrate (*M. musculus*) that exhibited a lethal phenotype upon gene knock-down or knock-out. In addition, we gave eight points to targets with a unique KEGG pathway term, reasoning that they might represent indispensable ‘choke-points’ that would lead to a major pathway disruption if inhibited or knocked down. For the same reason, proteins with a unique InterPro protein annotation were given seven points.

#### Selection of potential targets based on annotation (panel 4)

The “Annotation” panel allows the user to select a subset of proteins with a particular annotation as predicted targets. The selection panel can be searched by simply typing keywords into it; for example, typing “kinase” lists all InterPro terms containing “kinase” and allows individual terms to be selected. Multiple terms can be selected and the check-box “All of selected” allows for the selection of proteins that have been assigned to all of the listed annotation terms. In contrast, the check-box “Any of the selected” selects all proteins with one or more of the listed terms, and the check-box “None of the selected” excludes any protein annotated with any of the terms listed. As we did not want to restrict our selection to a particular group or family of proteins, we chose the default selection (“All identifiers” and “Any of the selected”) for this field.

#### Drug-drug target associations and chemical properties of predicted ligands (panel 5)

To select both targets with or without associated compounds, we carried out two independent rounds of ranking. In the first round, we linked compounds to selected drug targets based on sequence similarity between parasite proteins and proteins deposited in the two databases, ChEMBL and DrugBank. The “Drugs” panel allows for further prioritisation of targets based on the presence and chemical properties of associated compounds in these databases. For both databases, we required each target to have at least one associated compound and assigned additional five points to the overall target score, provided that five or more such compounds were found, respectively. Additional information about these compounds was then used to further filter the results: for compounds in the ChEMBL database, we required the drug phase to be between phase II and phase IV [46]. The maximum number of rule-of-5 violations was set at zero. For DrugBank, we included only compounds that had the status “approved”.

In the second round, we excluded all targets that had compounds associated with them in either of the two databases by checking the “exclude” check-box for the “At least one associated compound in ChEMBL/DrugBank” features. All other parameters remained the same as in the first round of ranking. Using these settings, we ranked targets solely considering homology-based features and selected proteins that might represent potentially new drug targets for which no known ligands exist.

#### Prioritised proteins/targets (panel 6)

**Fig. 3:**
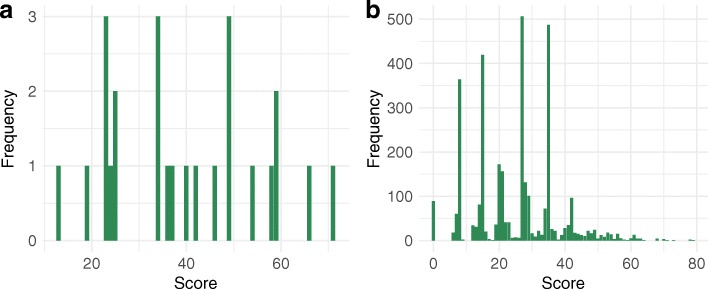
Score distributions for inferred *Schistosoma haematobium* drug targets. The distributions of scores for targets with associated drugs (*n* = 25; **a**) and those without associated drugs (*n* = 3402; **b**) are shown

The “Summary and Analysis” panel contains three main elements: a summary table (Table 1) that lists all weightings and ranking modes that have been set for individual features. All changes made to these parameters by an investigator can be reset to the parameters applied in this study by clicking the “Reset weightings” button. Clicking the “Calculate ranking” button reveals a range of additional elements in this panel: a histogram (Fig. 3a, b) summarises the number of proteins that satisfy the applied criteria and the distribution of scores for all predicted targets. The detailed score and annotation data can be downloaded as a tab-separated text file for a specified number of proteins (default: top 10% of all inferred proteins) or for all proteins by selecting a cut-off and clicking “Download protein data”. In addition, the applied ranking parameters can also be downloaded using the “Download ranking parameters” button. At the bottom of the panel, a table (Additional file 1: Table S1 and S2) lists all results, including the total score, information on protein annotation and scores for individual criteria. Criteria that are excluded or are required (which means they are not weighted) are not shown in the results list, as their values would be identical for all selected proteins. Entries in this table can be ordered according to the values within individual columns using the up- and down-arrows next to the column names, and can be searched using the text field at the top right of the table.

#### Predicted/prioritised drugs (panel 7)

The “Drug Results” panel contains two sub-panels, one for ChEMBL and one for DrugBank, and lists information on all associated compounds that satisfy the selection criteria chosen in the “Drugs” panel for each predicted target. In addition to summarising the properties of the resultant compound-target relationships, the ChEMBL result table contains patent information as well as web-links to data sheets of compounds and associated screening assays. Both result tables are searchable and can be downloaded by clicking the “Download ChEMBL/DrugBank data” button. For both databases, the number of targets for which associated compounds are shown can be selected (default: top 10% of all inferred targets). We investigated further the top five compounds from each of the two sub-panels.

## Results

### Predicted targets associated with drugs (‘repurposing’)

The filtering and ranking criteria applied in the first round yielded 25 potential targets that had one or more drugs associated with them (Fig. 3a; Additional file 1: Tables S1 and S3). Most of these targets (76%) had a score of < 50 (Fig. 3a). We selected the five targets that had the highest scores (58 to 71) for further assessment. Among them were a G protein-coupled receptor (MS3_07429), two protein kinases (MS3_03067 and MS3_07186), a “major facilitator superfamily” protein (MS3_09816) and a peptidase (MS3_02082). The latter two were excluded from the target list during the compound association process because, although compounds were linked to them, they did not meet requirements set for the drug phase and/or rule-of-5 violations. The three most highly ranked targets inferred were linked to 104 distinct compounds in the ChEMBL database (Additional file 1: Table S3). Of all 127 predicted associations, 77 compounds were linked to MS3_07429, whereas 27 and 23 compounds were associated with the two kinases, respectively. Of all predicted drugs, three compounds [CHEMBL490 (paroxetine), CHEMBL607707 (pelitinib), CHEMBL513909 (Bi-2536)] predicted to target one or more of the top three targets showed activity against schistosomes *in vitro* in previous studies [38, 47]. However, for all of these drugs the predicted targets differed from those reported earlier. For paroxetine, a G protein-coupled serotonin receptor was predicted to be the target, whereas Neves and colleagues [47] reported a serotonin transporter as the putative target. Similarly, the two targets [a SNF-related serine/threonine-protein kinase (SNRK) and a brain-selective kinase 2 (BRSK2)] predicted for the other two compounds were distinct from those reported earlier, i.e. a polo-like kinase 1 (PLK1) and an epidermal growth factor receptor (erbB1), respectively [38]. In the DrugBank database, 42 compounds were associated with MS3_07429, three with MS3_07186 and one with MS3_09816 (Additional file 1: Table S4). Of all 46 compounds inferred from DrugBank, ten were identical to those in the ChEMBL database, including paroxetine.

### Predicted targets for novel drugs (‘*de novo* discovery’)

In contrast, excluding all targets with associated compounds yielded 3402 potential targets (Additional file 1: Table S2). Despite this relatively high number of selected proteins (30.5% of the entire proteome), the score distribution was clearly skewed towards lower values, with the majority of predicted targets (*n* = 3034; 89.2%) having a score of ≤ 40. We selected the top five targets that had a score of ≥ 71 for further evaluation. Among them were two NADH-ubiquinone oxidoreductase subunits (MS3_05808 and MS3_02704), two ribosomal proteins (L1; MS3_01508 and L9; MS3_07928) and a protein annotated as a “dehydrogenase/reductase SDR family member 12” based on its closest homolog (11% sequence similarity; A6QP05) in the Swiss-Prot database. However, using InterProScan, this predicted target was annotated as a “transcription initiation factor IIF, alpha subunit” (IPR008851). Upon closer inspection, this protein and its closest homolog in Swiss-Prot showed sequence similarity in a section of the protein that represented a “NAD(P)-binding domain” (homology superfamily IPR036291), explaining the difference in annotation between the Swiss-Prot and InterPro databases.

## Discussion

Here, we established an improved approach for the prediction of potential drug targets in parasitic worms, and created an online application for the reproducible and customisable prioritisation of putative targets of small-molecule schistosomicidal agents in *S. haematobium*. Prediction and prioritisation are guided by information obtained from the analysis and comparison of genomic and transcriptomic data sets for *S. haematobium* and those of relevant related organisms. The customisable nature of the prioritisation approach might be beneficial to researchers wanting a means of prioritising drug targets of *S. haematobium* based on sequence- and compound-based criteria.

We presented one possible prioritisation strategy, as an example, and have given reasons for the selection of weightings, cut-offs and other parameters. Our prioritisation strategy might not necessarily reflect the way other researchers might wish to prioritise targets, based on their unique research questions and/or capacity to further investigate drug target candidates. For example, here, we gave eight points to potential targets for which an *F. hepatica* ortholog existed, reasoning that a novel target should be present in most or all trematodes of major socioeconomic importance (providing an increased financial incentive for companies to pursue drug development efforts). Another example relates to the cut-offs set for minimum similarity (80%) and minimum coverage (50%) for a sequence to be considered an ortholog. These values might be more stringent, depending on the judgements of, or considerations by, other investigators. Importantly, the interactive, online application readily allows for such adjustments to be made (and saved as a parameter file) *via* the user interface, thus enabling different prioritisation approaches and parameter settings to be compared or contrasted. Similarly, the criteria for the selection of compounds considered to be suitable candidates as anti-schistosomal (or trematocidal) drugs can vary markedly from investigator to investigator. The current platform provides a range of customisation options, including setting requirements for the approval status of a drug, patents, previous screening results and/or (bio-)chemical properties.

The ability to adjust the ranking and prioritisation, according to the perceived importance of individual properties of a target or drug, is considered to represent a ‘transparent’ way to select targets for future investigations. However, although selection parameters can be justified, they are often arbitrary and depend on a researcher’s subjective notion of the importance of individual criteria, as indicated in earlier studies [16, 34]. In practice, this means that investigators tend to adjust their parameters, so that the number of predicted targets match their desired outcome by heavily weighting or filtering according to the most selective predictors. Choosing stringent filtering options will reduce the number of targets, reducing or eliminating the need to choose a cut-off score. In contrast, assigning weightings to individual features/categories does not initially exclude any proteins, thus resulting in a large number of targets, requiring a subsequent selection of a small number of the highest-ranked targets for further pursuit. Importantly, the ability to compare and contrast a range of prioritisation scenarios, in association with experimental validation, can objectively assess the validity of the perceived importance of individual properties, and thus enhance future *in silico* prioritisation. Experimental validation should also inform about the reliability of different selection and ranking features and parameters, by showing how changes to the weighting of individual features can affect the variability of the resultant ranking, and thus revealing features that have the greatest effect(s) on the distribution of scores.

In this context, the visual representation of clusters of categories for all proteins investigated (Fig. 1) is a useful tool to gauge the number of genes/proteins filtered when a particular feature is set to “required”, and how weightings given to individual categories affect the overall score. This representation of features also summarises, visually, the relationships among and/or dependencies of features. For example, prioritising proteins which are conserved in primary sequence among flatworms but not present in mouse and other model organisms ranks these molecules in the lower third of the heatmap (Fig. 1) most highly; in contrast, additionally filtering these proteins for those having compounds linked to them removes most targets from the results, as evidenced in the last four columns of the lower section of the heatmap (Fig. 1). On the other hand, selecting proteins that associate with known drugs is more likely to prioritise proteins that are conserved among all species compared (including mouse and human), which cluster in the upper two thirds of the heatmap (Fig. 1).

We emphasise that the present study presents a tool for drug target prioritisation, but does not determine which features are verifiably the most informative for determining viable or genuine drug targets. Such an endeavour would require the experimental validation of a range of different prioritisation scenarios *via* drug screening, biochemical investigations and/or functional genomics, which is beyond the scope of this bioinformatic investigation. However, importantly, the present application does allow for different scenarios to be simulated, and thus might underpin the selection of features that consistently enrich viable drug targets among the most highly ranked putative targets.

A comparison of the present approach to previous efforts of computationally prioritising drug targets, guided by genomic and transcriptomic data, suggests that there is merit in ‘data-driven’ selection of potential targets. For example, a similar ranking approach inferred a number of protein kinases as putative drug targets in *H. contortus* and linked them to small molecules that were later verified as promising lead candidates in an *in vitro* study [48]. However, this finding is somewhat anecdotal and requires a large-scale, comprehensive evaluation to show that the present ranking approach produces a statistically significant enrichment of promising targets and/or compounds. Additionally, some of the ranking features applied to drug target prediction in *H. contortus* might have performed well due to a relatively close genetic similarity to *C. elegans*. Whether the present, enhanced approach performs equally well for flatworms remains to be assessed in future evaluations. In this context, the inference of evolutionarily distant orthologs in flatworms might be achieved by employing a recently-developed taxon sampling approach [49], which can overcome long evolutionary distances and identify putative hidden orthologs by employing a transitive homology approach [49].

In addition to such challenges, differences in methodology, data sets and applied parameters of *in silico* drug target prediction approaches make it difficult to directly compare studies, even for predictions made for the same species, as indicated earlier [50]. For example, at the time of *in silico*-drug target prediction efforts carried out for *S. mansoni* or *S. haematobium* [10, 11, 14], genomic and transcriptomic data were largely unavailable for other flatworm species of socioeconomic importance (e.g. *C. sinensis*, *O. viverrini*, *F. hepatica*). Data for these species are now accessible, and integrating them in the prioritisation strategy applied here had a substantial effect on the score distribution (Table 1 and Fig. 1). Additionally, the quality of draft genomes is continually evolving, mainly due to concerted curation efforts by individual curators and the scientific community [23, 51, 52]. Taken together, the differences in the amount and quality of data that were integrated into the prioritisation approaches is likely to have led to some discrepancies in the prediction of drug targets between the present and earlier studies. In the future, the assembly of novel and curation of existing draft genomes and their improved annotation should enhance the accuracy with which we are able to infer the function of proteins and the essentiality of genes, thus making drug target prediction more reliable.

Despite current challenges, some overlaps were apparent in the target and compound lists between this and earlier studies. For example, most studies reporting putative drug targets in schistosomes, including the present investigation, consistently rank highly enzymes involved in phosphorylation-dependent signalling, such as kinases [10, 12, 14] and phosphatases [11]. Other examples of commonly prioritised targets include proteases [10, 14], proteins and enzymes with roles in G protein signalling [10, 14], reductases [14, 50] and transporters [10, 14, 50]. For transporters and kinases, in particular, there is accumulating evidence that they are amenable to targeting by small molecular drugs in schistosomes [38, 47, 53, 54]. The finding that a number of drugs predicted here and in a previous study [12] (including dasatinib, imatinib, pelitinib, Bi-2536 and paroxetine) have known anti-schistosomal activity *in vitro* [38, 47, 53, 55] instils confidence that a bioinformatics approach identifies compounds that merit further pursuit. Interestingly, our approach did not predict praziquantel as a drug in either of the two drug databases. Although ChEMBL contains 264 results for binding assays (“B”) for praziquantel (CHEMBL976), no activity values are recorded. Thus, in our approach (i.e. selecting compounds for which assays are recorded that report an activity value of ≤ 10 mM), praziquantel entries were ‘filtered out’ and, thus, are absent from the candidate list, irrespective of the ranking strategy. For DrugBank, the target annotation for praziquantel (DB01058) is “schistosome calcium ion (Ca^2+^) channels”, but no sequence data is associated with this annotation, thus preventing a link between praziquantel and a target sequence. These findings reflect the need for the curation and/or correct submission of (meta-)data to the ChEMBL and DrugBank databases, in order to improve the accuracy and sensitivity of predictions reliant on such data.

Additionally, there should be a focus on expanding the capabilities of the present online application by including protein structure modelling (cf. [56, 57]). Such an approach, combined with refined structural prediction strategies, such as the comparison of binding pockets inferred for different proteins and the identification of particular ‘sub-pockets’ [58, 59] followed by virtual screening and/or docking approaches [60], might be employed to indicate the binding mode of a ligand to a predicted structural element, without requiring prior evidence of a target-compound relationship. A limitation of such approaches is the lack of experimentally determined three-dimensional (crystal) structures for most proteins of parasitic worms and a reliance on the computational modelling based on structural homology [57]. This aspect highlights the need for structural investigations of proteins of parasitic worms to assist in assessing their potential as drug targets. Nevertheless, the proposed computational modelling approach would enable the ‘pre-screening’ of large compound libraries against predicted target structures (cf. [61, 62]), possibly representing a cost- and time-efficient step prior to costly and time-consuming *in vitro* screening or functional genomics experiments.

For schistosomes, there are numerous reports of the successful application of RNAi [63–66] and lentiviral-based knockdown [67, 68], and CRISPR/Cas-9-based approaches are currently undergoing development [69]. Extending the present study, one or more of these functional genomics tools could be used to study the roles and essentiality of predicted targets [66, 70]. Such experiments would provide crucial evidence to gain confidence about essentiality predictions using computational means. In addition to knockdown experiments, the use of small-molecular chemicals to elicit lethal or sub-lethal phenotypes, would also have merit, and could support essentiality and drug target predictions. In this context, compounds that have been linked to five prioritised targets in the present study appear to represent prime candidates for *in vitro*-testing against schistosomes using established methods [53, 71, 72]. Subsequently, RNA-sequencing and proteomic analyses of treated, untreated and/or knocked-down schistosomes might provide some additional support to the target-compound relationships predicted here, could inform about possible limitations of computational predictions and would likely stimulate areas worthy of improvement.

## Conclusions

The online application developed here represents an interactive, customisable, expandable and reproducible drug target prioritisation and ranking approach that, we believe, should be useful for the prediction and prioritisation of drug targets in schistosomes and, after expansion, other species of parasitic worms. Using the established online application, we have predicted ten targets that can now be evaluated using functional genomics tools and/or small molecules, to establish whether they are indeed essential for parasite survival in *S. haematobium*.

## Additional file

Additional file 1: **Table S1.** Predicted targets in *Schistosoma haematobium* that have compounds associated with them, their annotation and individual feature scores/weightings. **Table S2.** Predicted targets in *Schistosoma haematobium* without associated drugs, their annotation and individual feature scores/weightings. **Table S3.** ChEMBL compounds associated with the top five predicted drug targets in *Schistosoma haematobium*, their chemical properties and drug information. **Table S4.** DrugBank compounds associated with the top five predicted drug targets in *Schistosoma haematobium*, their chemical properties and drug information. (XLSX 234 kb)

## Abbreviations

BRSK2: Brain-selective kinase 2
CRISPR: Clustered regularly interspaced short palindromic repeats
IC50: Half-maximal inhibitory concentration
Kd: Dissociation constant
KEGG: Kyoto Encyclopedia of Genes and Genomes
Ki: Inhibitor constant
KO: KEGG orthologous gene
MDA: Mass drug administration
MDDR: Molecular Design Limited (MDL) Drug Data Report
NAD(P): Nicotinamide adenine dinucleotide (phosphate)
NADH: Nicotinamide adenine dinucleotide + hydrogen
NTD: Neglected tropical disease
PLK1: Polo-like kinase 1
RNA: Ribonucleic acid
RNAi: RNA interference
SDR: Short-chain dehydrogenases/reductases family
SNRK: SNF (sucrose non-fermenting)-related serine/threonine-protein kinase
